# A retrospective analysis of the definitive management of open talus fractures at a major trauma centre, comparing ORIF to FUSION: cohort study and audit of BOAST 4 guidelines

**DOI:** 10.1007/s00590-022-03204-3

**Published:** 2022-01-14

**Authors:** Andrew Kailin Zhou, Eric Jou, Reece Patel, Faheem Bhatti, Nishil Modi, Victor Lu, James Zhang, Matija Krkovic

**Affiliations:** 1 Department of Trauma and Orthopaedics, Addenbrookes Major Trauma Unit, Cambridge University Hospitals, Cambridge, UK; 2 School of Clinical Medicine, University of Cambridge, Cambridge, CB2 0SP UK

**Keywords:** Talus, Tibiocalcaneal fusion, Bone transport, Internal fixation

## Abstract

**Purpose:**

Open talus fractures are notoriously difficult to manage, and they are commonly associated with a high level of complications including non-union, avascular necrosis and infection. Currently, the management of such injuries is based upon BOAST 4 guidelines although there is no suggested definitive management, and thus, definitive management is based upon surgeon preference. The key principles of open talus fracture management which do not vary between surgeons are early debridement, orthoplastic wound care, anatomic reduction and definitive fixation whenever possible. However, there is much debate over whether the talus should be preserved or removed after open talus fracture/dislocation and proceeded to tibiocalcaneal fusion.

**Methods:**

A review of electronic hospital records for open talus fractures from 2014 to 2021 returned fourteen patients with fifteen open talus fractures. Seven cases were initially managed with ORIF, and five cases were definitively managed with FUSION, while the others were managed with alternative methods. We collected patient’s age, gender, surgical complications, surgical risk factors and post-treatment functional ability and pain and compliance with BOAST guidelines. The average follow-up of the cohort was 4 years and one month. EQ-5D-5L and FAAM-ADL/Sports score was used as a patient reported outcome measure. Data were analysed using the software PRISM.

**Results:**

Comparison between FUSION and ORIF groups showed no statistically significant difference in EQ-5D-5L score (*P* = 0.13), FAAM-ADL (*P* = 0.20), FAAM-Sport (*P* = 0.34), infection rate (*P* = 0.55), surgical times (*P* = 0.91) and time to weight bearing (*P* = 0.39), despite a higher proportion of polytrauma and Hawkins III and IV fractures in the FUSION group.

**Conclusion:**

FUSION is typically used as second line to ORIF or failed ORIF. However, there is a lack of studies that directly compared outcome in open talus fracture patients definitively managed with FUSION or ORIF. Our results demonstrate for the first time that FUSION may not be inferior to ORIF in terms of patient functional outcome, infection rate and quality of life, in the management of patients with open talus fracture patients. Of note, as open talus fractures have increased risks of complications such as osteonecrosis and non-union, FUSION should be considered as a viable option to mitigate these potential complications in these patients.

## Introduction

Open talus fractures are rare but serious fractures, commonly associated with a variety of complications such as non-union, avascular necrosis and infection producing far less favourable outcomes for the patient [1–3]. In particular for talar neck fractures, Hawkins III and IV fractures (with fracture displacement) are associated with poor prognosis compared to Hawkins I and II fractures (minimal or no displacement) [4]. The initial management of such injuries is based upon BOAST 4 guidelines [5]. The current definitive management is based upon surgeon preference; however, the key principles of open talus fracture management which do not vary between surgeons are early debridement, orthoplastic wound care, anatomic reduction and definitive fixation [6]. There is much debate over whether the open reduction internal fixation (ORIF) or removal of the talus and proceeding to tibiocalcaneal fusion (FUSION) is the best initial definitive management [7–9] for Hawkins III and IV fractures regardless if they are open or closed.

Many patients post-ORIF of talus fracture will experience pain and will later develop secondary hindfoot arthritis despite an anatomical reduction [10]. Complications of ORIF of a talus fracture include infections, non-union, avascular necrosis, ankle and subtalar osteoarthritis which all contribute to the patient having worse outcomes and further procedures [2, 3, 11]. However, an alternative to ORIF of the talus is removal of talus with proceeding to a tibiocalcaneal fusion [8, 12, 13]. This method is currently frowned upon by many who believe talus fracture should be managed restoring the natural anatomy and preserving the joint congruity [6, 14]. The accurate restoration of joint congruity is to minimise long-term degenerative changes; however, ORIF patients can experience prolonged pain and decreased mobility, despite an appropriate pain control [10].

Currently, one of the options for management of failed ORIF of the talus is a tibiocalcaneal fusion [12]. Gait analysis in patients with FUSIONS will show a shortened stride, but normal cadence and velocity [15]. However, FUSION is currently only indicated in when ORIF fails and in severe cases of open talus fractures [12]. There is no strong evidence to suggest what the long-term effects of tibio-talar fusion are on the mid-foot joints of the injured foot.

The aim of this study is to analyse and compare the outcomes of patients who have had ORIF and patients who have had FUSION of the talus for the definitive management of open talus fractures.

## Method

We have registered this project under as an audit with the identification number of 3932 and project reference number of 9932. A retrospective cohort study was conducted at a major trauma centre in the UK. The hospital’s electronic patients’ records were reviewed from January 2014 to July 2021 for open talus fractures in skeletally mature patients. Exclusion criteria encompassed patients who were deceased at the time of review and a follow-up time of less than 24 months. There were fourteen patients and fifteen open talus fractures; one patient had bilateral open talus fractures (Table 1). The patient population was homogenous as majority of patients were from a traumatic cause with the 4 patients due to road traffic accident (Table 2). The mean age of admission was 45, and 29% (4/14) were female (Table 1). Eleven (9/15, 60%) of patients were under the definition of polytrauma (Table 3), a term used for severely injured patients associated with two or more severe injuries in at least two areas of the body [16]. Both classifications of talus fractures were recorded: Gustilo–Anderson score and anatomical classification [17]. Assessment using the Gustilo–Anderson classification system revealed that the majority of our cohort was classified as 3B (60%, 9/15 fractures), followed by 3A (33.3%, 5/15 fractures) [17, 18] (Table 3). Hawkins classification for talus neck fracture was used for the anatomical classification if the fracture was outside of the talar neck, and we reported the anatomical region of the talus which was fractured [18].

**Table 1:** Patient characteristics of patients with open talus fractures

Characteristic	Number of patients
Total fractures	14
Male (%)	10 (71%)
Age (mean)	46
Age (median and range)	44.5 (18–78)

**Table 2:** Causes of injury

	Hawkins 1 and 2		Hawkins 3 and 4		
	ORIF (*N* = 3)	FUSION (*N* = 1)	ORIF (*N* = 2)	FUSION (*N* = 4)	ORIF + FUSION (*N* = 1)
Road traffic accident	1	1	1	2	0
Crush injury	1	0	0	0	0
Sport injury (Rugby)	1	0	0	0	0
Fall	0	0	0	0	1
Suicide attempt	0	0	0	2	0
Assault	0	0	1	0	0

Ankle X-rays were analysed before the surgery to obtain the classification and confirm the fracture. The patient’s age, gender, surgical complications, surgical risk factors and post-treatment functional ability, pain, the localisation of such pain and data regarding to the BOAST 4 guidelines compliance were extracted through the hospital’s EPIC patients record system. Operative American Standard of Anaesthesiologists' (ASA) score was recorded with the patients’ comorbidities (Tables 3 and 4). As no bespoke scoring system currently exists quantifying outcomes for open talus fracture management, the Foot and Ankle Measure (FAAM) was adopted in this study due to incorporating functional and anatomical factors [19]. There are two parts of the FAAM questionnaire; we utilised both the activities of daily living (ADL) and sport section. Four cases with lower limb amputations were not included in the FAAM scoring analysis because they did not have an ankle making FAAM not applicable. Patient outcomes were analysed through recording data from the patient database and further supplementing with patient reported outcome measures. We assessed the post-operative quality of life using the 5-level EQ-5D (EQ-5D-5L) score [20]. FAAM and EQ-5D-5L scores were collected through phone calls due to restrictions from COVID-19; this was common practice during COVID-19 [21]. 88% (15/17) of the cohort responded to the questionnaire through a combination of emails and phone calls. Two patients did not respond despite email and three phone calls on three separate occasions. Data were analysed using the software PRISM.

**Table 3:** Comorbidities of the patients

Comorbidities of patients	Frequency
Hypertension	3
*BMI > 25	5
Smoking/Vaping	1
Alcohol excess	1
Anxiety/Depression	2
Temporal arteritis	2
Arrhythmias	2
Type 1 diabetes mellitus	1
Hypothyroidism	1
Asthma	1
Autoimmune hepatitis	1

*body mass index (BMI)

**Table 4:** Patient details of fracture and method of fixation, *Open reduction internal fixation (ORIF), tibiocalcaneal fusion (FUSION)

Patient numbers	Age on admission	Gender	Fracture classification (Gustilo–Anderson)	Fracture classification (Anatomical/Hawkins classification)	Method of definitive fixation	Cause of injury	Polytrauma?	Amputation?
1	28	Male	3A	Hawkins I	ORIF	Crush accident	Yes	Yes
2	37	Male	3A	Hawkins II	Internal fixation with K-wires	Fall from a horse	No	No
3	34	Female	3B	Hawkins II	FUSION	Road traffic accident	Yes	No
4	67	Male	3B	Hawkins II	ORIF	Road traffic accident	Yes	No
5	44	Female	2	Hawkins I	ORIF	Sport- Rugby	No	No
6	39	Male	3B	Hawkins I	Soft tissue management	Road traffic accident	Yes	No
7	55	Male	3B	Hawkins III	FUSION	Road traffic accident	No	No
8	32	Male	3B	Hawkins IV	FUSION	Road traffic accident	Yes	No
9	46	Male	3B	Hawkins IV	ORIF	Road traffic accident	Yes	No
10	18	Male	3A	Hawkins IV	ORIF	Assaulted	No	No
11**	**78**	**Female**	**3A**	**Hawkins III**	**Fusion**	**Suicide attempt**	**Yes**	**Yes**
12**	**78**	**Female**	**3B**	**Hawkins III**	**Fusion**	**Suicide attempt**	**Yes**	**No**
13	63	Female	3B	Hawkins III	ORIF + FUSION	Fall of 3 m	Yes	No
14	45	Male	3A	Talar dome	ORIF	Road traffic accident	No	No
15	56	Male	3B	Severe comminution	Amputation	Gunshot	No	Yes

The bold values are to signify that the fractures are from the same patient

** Patients 11 and 12 are the same patient who had bilateral open talus fractures

## Results

**Fig. 1:**
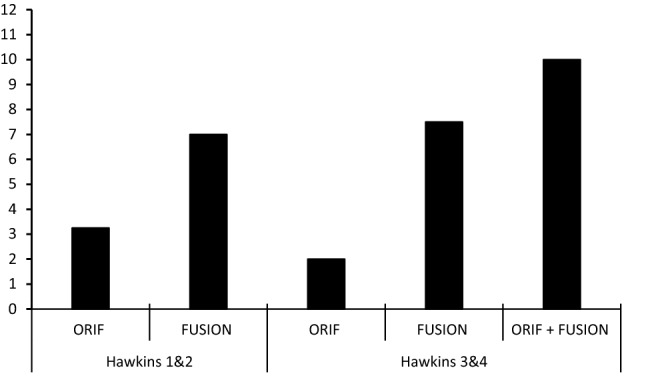
Average number of procedures required for definitive treatment

All of cases reported orthoplastic involvement, 100% of wounds were managed initially with saline-soaked gauze and an occlusive film, 100% of cases reported that patients received expected functional recovery and rehabilitation advice, and 100% of cases recorded debridement although only 60% of cases reported debridement using fasciotomy lines. However, only 27% of cases reported prophylactic antibiotics within one hour of injury and definitive soft tissue coverage within 72 h was only achieved in 27% of cases. Seven patients required soft tissue coverage, and definitive soft tissue coverage was achieved with a mean of 9.7 days after the injury. Three of the patients who had definitive soft tissue coverage were FUSION patients, and on average, the definitive soft tissue coverage was achieved 20.7 days after injury. Four ORIF patients went on to have definitive soft tissue fixation, on average 4.4 days after injury. On average, application of external fixator was applied 14.4 days after initial debridement in FUSION patients. Two of the fractures managed with FUSION used Oxbridge tibiocalcaneal fusion nails, while for the other 3 FUSION patients, the implant was not documented. ORIF was performed, on average, 6.9 days after initial debridement. Patient 13 underwent ORIF 4 days after initial debridement which was later followed by extrusion of the talus 30 days later (Table 5). 450 days after initial ORIF, patient 13 proceeded to have FUSION with the Oxbridge tibiocalcaneal fusion nail (Fig. 1).

The main trauma cause was road traffic accidents. Suicide attempts were the next most common cause of open talus fractures, while the rest consisted of rugby injury, crush injury and assault (Table 2). We recorded the comorbidities of patients prior to injury and the average ASA score of the cohort was 2. Over a third of the cohort was overweight, and 20% of the patients were diagnosed with previous hypertension (Table 4). Fractures 11 and 12 were from a patient who suffered from bilateral open talus fractures which was a result of a suicide attempt and had a history of anxiety and depression (Tables 4 and 5).

Six of the fractures were treated with ORIF as the initial definitive management, five received FUSION, and one had ORIF first followed by FUSION, while the rest were managed by other methods (Table 3). More polytrauma patients were treated with FUSION (80%, 4/5 fractures) compared to ORIF (50%, 3/6 fractures). On average, five procedures were required to complete the treatment. All patients with more than two procedures required multiple debridement or vacuum dressing changes. One case of ORIF subsequently required amputation due to a combination ORIF failing and gangrene, and this occurred after he was transferred to another hospital. One patient developed avascular necrosis prior to definitive fixation; therefore, this patient was managed with a FUSION initially.

The average follow-up time for our patient reported outcome measures is 231 weeks (4 years 1 months) with a range of 115 weeks and 335 weeks (2 years 2 months and 6 years and 5 months, respectively) (Table 5). The average EQ-5D-5L index score was 0.543 (*N* = 14 patients), and there was no statistically significant difference between FUSION and ORIF (Mann–Whitney test, *P* = 0.13; ORIF median = 0.743, *n* = 5; FUSION median = 0.620, *n* = 5). Average FAAM-ADL and FAAM-sport score was 57% and 35%, respectively. When patients were asked to rate their current level of function during your usual activities of daily living (from 0 to 100), the average was 31.25 (range = 0–75) and this was the same for ankle function during usual sports-related activities. Those that were amputated were not included in the qualitative and quantitative synthesis for the FAAM scores. While there may be a potential trend, there was no statistical difference (Mann–Whitney test, *P* = 0.20) in the FAAM-ADL between the two groups, indicating similar functional outcomes in patients treated with ORIF (median 75.5, *n* = 4) or FUSION (median 38.5, *n* = 4). We did not include patient 1 (ORIF with subsequent FUSION) in the statistical analysis as the patient had undergone both procedures. Accordingly, there was also no significant difference in FAAM-sport score (*P* = 0.34) between patients treated with ORIF (median 45.5, n = 4) or FUSION (median 21.5, *n* = 4).

Overall, 7 cases (47%) reported deep surgical site infection after surgery; in one case the infection did not resolve and required amputation; however, in six cases (6/7, 85%) bone infections were resolved with combinations antibiotics. Three patients developed surgical site soft tissue infection, and two (2/3, 67%) cases were resolved with combinations of antibiotics. The incidence of infection (either type) in patients managed with ORIF and FUSION was 50% (3/6 fractures) and 80% (4/5 fractures), respectively (Table 6). Direct comparison of the infection rates between open talus fracture patients managed with ORIF or FUSION revealed no statistical difference (Fisher’s exact test, *P* = 0.55), for all parameters investigated including bone infections, and surgical site wound infections, either assessed alone or pooled. Other parameters such as surgical procedure time (Mann–Whitney test, *P* = 0.91, ORIF median = 258.5 min, *n* = 6; FUSION median = 251 min, *n* = 4) and time to weight bearing after surgery (Mann–Whitney test, *P* = 0.39, ORIF median = 47.0 days, *n* = 5; FUSION median = 64 days, *n* = 3) also revealed no statistical difference between patients managed with ORIF or FUSION. In the time to weight bearing analysis, patients that subsequently received an amputation were excluded.

**Table 5:** Complications after operation for patients with open talus fractures

	Hawkins 1 and 2		Hawkins 3 and 4		
	ORIF (*N* = 3)	FUSION (*N* = 1)	ORIF (*N* = 2)	FUSION (*N* = 4)	ORIF + FUSION (*N* = 1)
Bone and joint infection	1	0	0	4	1
Surgical site infection	1	0	1	0	1
Non-union	0	0	0	0	1
Avascular necrosis	0	0	0	0	0
Valgus deformity	0	1	0	3	1
Fixed flexion deformity	0	0	0	0	0
Ankle osteoarthritis	1	0	0	0	0
Amputation	0	0	0	1	0
Intra-operative bleeding	0	0	1	0	0

**Table 6:** Patient outcomes

Patient number	Follow-up time (Days)	Fracture classification (Anatomical/Hawkins classification)	Method of definitive fixation	“How would you rate your current level of function?” (FAAM)	FAAM-ADL (%)	FAAM-Sport (%)	EQ-5D-5L index score	“How good or how bad is your health today from a scale of 0–100?” (EQ-5D-5L)*
1	1419	Hawkins I	ORIF	***N/A	***N/A	***N/A	0.743	40
2	879	Hawkins II	Internal fixation with K-wires	Nearly Normal	95%	78%	0.796	95
3	1642	Hawkins II	FUSION	Severely Abnormal	21%	0%	-0.184	30
4	2102	Hawkins II	ORIF	Abnormal	37%	13%	0.813	75
5	1068	Hawkins I	ORIF	Nearly Normal	89%	50%	0.837	80
6	1398	Hawkins I	Soft tissue management	Abnormal	37%	25%	0.345	40
7	1193	Hawkins III	FUSION	Nearly Normal	61%	44%	0.548	70
8	2179	Hawkins IV	FUSION	Abnormal	38%	25%	0.620	50
9	2249	Hawkins IV	ORIF	Abnormal	77%	41%	0.598	65
10	2347	Hawkins IV	ORIF	–	–	–	–	–
11**	1855	Hawkins III	Fusion	***N/A	***N/A	***N/A	0.665	70
12**	1855	Hawkins III	Fusion	Nearly Normal	39%	18%	0.665	70
13	808	Hawkins III	ORIF + FUSION	Nearly Normal	26%	13%	0.185	50
14	1292	Talar dome	ORIF	Nearly Normal	74%	75%	0.636	75
15	2025	Severe comminution	Amputation	***N/A	***N/A	***N/A	1.000	50

* (Best possible health = 100, worst possible health = 0), ** Patients 11 and 12 are the same patient who had bilateral open talus fractures, ***Those who were amputated during the FAAM score data collection were not included in the FAAM score analysis

In our cohort of patients with open talus neck fractures (Table 3), there was a greater proportion of Hawkins type III or type IV fractures (53.8%, 7/13 fractures) compared to Hawkins type I or type II. In the present study, of the Hawkins I and II fractures managed with ORIF or FUSION, 75% (3/4) were managed with ORIF, while 25% (1/4) was managed with FUSION. For Hawkins I and II, ORIF patients had an average EQ-5D-5L of 0.798 (*N* = 3), while FUSION patient had an EQ-5D-5L score of -0.184 (*N* = 1). A similar trend was observed with FAAM-ADL and FAAM-sport, 63 and 31.5, respectively, for ORIF (*N* = 3) and 21 and 0, respectively, for FUSION (*N* = 1). For Hawkins I and II fractures managed with ORIF, 33.3% (1/3) had infection, and 33.3% (1/3) had ankle osteoarthritis. Only one patient in this category was managed with FUSION and developed valgus deformity. On the other hand, for Hawkins III and IV fractures, 28.6% (2/7) were managed with ORIF, while 57.1% were managed with FUSION (4/7). One patient (14.3%) received ORIF initially followed by FUSION and was not included in the subsequent analysis. One of the Hawkins III and IV fractures required FUSION due to avascular necrosis. For Hawkins III and IV, the ORIF patient has an EQ-5D-%L score of 0.598 (*N* = 1), while FUSION patients had an average EQ-5D-5L of 0.611 (*N* = 3, as patient numbers 11 and 12 are the same patient). In Hawkins III and IV, the FAAM-ADL and FAAM-sport score was 77 and 41, respectively, for ORIF (*N* = 1), and 46 and 29, respectively, for FUSION (*N* = 3). 50% (1/2) of Hawkins III and IV fractures managed with ORIF developed infection, and 50% (1/2) had intra-operative bleeding. For Hawkins III and IV fractures managed with FUSION, all were associated with infection (4/4), 75% (3/4) had valgus deformity and 25% (1/4) received subsequent amputation. There was one case of non-union due to failure of ORIF (also showed limb length discrepancy), and this was subsequently successfully managed with a FUSION (Table 3).

## Discussion

Open talus fractures are serious injuries that typically follow high-energy traumatic events similar to our cohort [22]. Only 6.7% of had a history of diabetes, and this was similar to another study based in the UK (Table 4) [23]. It is estimated that approximately 20–25% of talus fractures are open fractures and commonly associate with adverse complications including non-union, avascular necrosis, post-traumatic arthritis and infection [22, 24–27]. Together with the lack of consensus guidelines on the definitive treatment of open talus fractures, management has been particularly challenging. While FUSION is typically recommended to be second line to ORIF, to our knowledge, there are no studies to date that directly compared the outcome of patients with open talus fractures after definitive management with ORIF or FUSION. Additionally, there are other surgical management options of open talus fractures such as internal fixation with K-wires or soft tissue management only, and these were used when ORIF or FUSION was declined. Currently, the literature mainly consists of ORIF, with FUSION being the next most common, while there are very little on other managements of open talus fractures. To our knowledge, the management of Hawkins I and II talus fractures are typically with ORIF, while Hawkins III and IV are usually managed with FUSION. Nevertheless, there are still some discrepancies among the surgeons at a major trauma centre and ultimately it will be based upon surgeon’s discretion.

In this study, there was no difference in open talus fracture patients treated with FUSION or ORIF, in terms of functional outcome (FAAM), quality of life (EQ-5D), surgical procedure time, infection rate, and time to weight bearing, despite the higher proportion of polytrauma, and Hawkins type III and IV fractures, in patients treated with FUSION compared to ORIF. These results suggest that despite the FUSION cohort having a higher proportion of fractures with poor prognosis, the outcome is similar to management of less severe fracture classifications with ORIF, indicating that early FUSION may be considered as a suitable alternative in managing polytraumatic Hawkins III and IV open talus fracture patients. In addition, while FUSION was proposed to be associated with increased risk of infection compared to ORIF [28], we did not find a statistically significant difference in infection rate between fractures managed with ORIF or FUSION in our study. To prevent further infection, we fused the joint only when the soft tissue has healed (4–5 weeks after soft tissue cover), the rationale being that if the soft tissue has not healed, the external fixator will restrict access and infection will contaminate the metalwork.

A recent study that did not discriminate between open and closed talus fractures found that 20% of talus neck fractures were Hawkins type I (30/150 fractures), 44.7% were type II (67/150), 35% were type III (53/150), and there were no type IV fractures [29]. Another similar study which also included both open and closed fractures found 17.9% (5/28 fractures) were Hawkins type III fractures, and there were no patients with Hawkins type IV fractures, while the rest were either type I or type II fractures [30]. In our cohort of patients with open talus neck fractures, there was a greater proportion of Hawkins type III or type IV fractures (53.8%) compared to these studies. As the risk of avascular necrosis is much higher in Hawkins type III and type IV fractures compared to type I and type II [4], these results suggest that open fractures are at higher risk of avascular necrosis compared to closed talus fractures. Given that patients with open talus fractures may have higher risks of avascular necrosis, which is also reported by others [24], FUSION could be a suitable option in these patients as it by definition removes the risk of avascular necrosis, while potentially resulting in similar outcomes to ORIF.

There are few reports that specifically assess open talus fractures, due to their rarity, resulting in a relative lack of understanding of the patient characteristics that sustain these injuries. To our knowledge, the present study is the first to assess FAAM and EQ-5D-5L scores specifically in open talus fracture patients, and as expected, the median FAAM-ADL and FAAM-sport score was lower in our cohort of open talus fractures (57 and 35, respectively), compared to another study on patients with lateral process talar fractures, where both open and closed fractures were included (FAAM-ADL = 89, FAAM-sport score = 77) [31]. A recent study by Liu et al. analysed 51 patients at a level one trauma centre and provided important insight on the epidemiology of open talus fractures [22]. In that study, the majority of open talus fracture patients were male (86.3%, 44/51 patients), which is largely similar to our cohort being predominantly males (71.4%, 10/14 patients). This suggests that there is gender bias towards males in patients that sustain open talus fractures and is consistent with previous reports that did not discriminate between open and closed talus fractures, where male patients also predominate [32]. We found that road traffic accidents are the most common cause of open talus fractures, accounting for almost half of all cases (46.7%, 7/15 fractures), followed by falling from height, which included all the suicide attempts (26.7%, 4/15 fractures). Accordingly, Liu et al. also found road traffic accidents (41.2%, 21/51 patients) and falling from height (37.3%, 19/51 patients) to be the first and second most common causes of open talus fractures, respectively (Table 2) [22]. Both in our cohort, and Liu et al., found that Gustilo–Anderson classes 3A and 3B are the most common classification in open talus fracture patients. Importantly, the strikingly high degree of concordance between our cohort and Liu et al. indicates that that these observations may be representative of the general population of patients with open talus fractures.

## Conclusion

To conclude, while FUSION is typically used as second line to ORIF in the contemporary setting, there are a lack of studies that directly compared outcome in open talus fracture patients definitively managed with FUSION or ORIF. Our results demonstrate for the first time that FUSION may not be inferior to ORIF in terms of patient functional outcome, infection rate and quality of life, in the management of patients with open talus fractures. Of note, as open talus fractures, along with Hawkins III and IV fractures, have increased risks of complications such as osteonecrosis and non-union, FUSION should be considered as a viable option to mitigate these potential complications in these patients on long term. Important limitations of this study include the limited sample size, which is due to the rarity of open talus fractures. Furthermore, measurements of various parameters (e.g. FAAM, EQ-5D, surgical time, infection rate and time to weight bearing) and the demographics of open talus fracture patients are provided in this study, which can be used for comparison in future studies.

## Data Availability

Data were presented in article.
